# Apocynin Attenuates Diabetes-Induced Skeletal Muscle Dysfunction by Mitigating ROS Generation and Boosting Antioxidant Defenses in Fast-Twitch and Slow-Twitch Muscles

**DOI:** 10.3390/life12050674

**Published:** 2022-05-01

**Authors:** Sarai Sánchez-Duarte, Rocío Montoya-Pérez, Sergio Márquez-Gamiño, Karla S. Vera-Delgado, Cipriana Caudillo-Cisneros, Fernando Sotelo-Barroso, Luis A. Sánchez-Briones, Elizabeth Sánchez-Duarte

**Affiliations:** 1 Instituto de Investigaciones Químico-Biológicas, Universidad Michoacana de San Nicolás de Hidalgo, Francisco J. Mújica s/n, Col. Felicitas del Río, Morelia 58030, Michoacán, Mexico; 1315649c@umich.mx (S.S.-D.); rmontoya@umich.mx (R.M.-P.); 2 Departamento de Ciencias Aplicadas al Trabajo, Universidad de Guanajuato Campus León, Eugenio Garza Sada 572, Lomas del Campestre Sección 2, León 37150, Guanajuato, Mexico; smgamino@fisica.ugto.mx (S.M.-G.); ksvera@ugto.mx (K.S.V.-D.); ccaudillo@ugto.mx (C.C.-C.); f.sotelo@ugto.mx (F.S.-B.); biol.luis.22@gmail.com (L.A.S.-B.)

**Keywords:** diabetes, skeletal muscle, NADPH oxidases, apocynin, oxidative stress

## Abstract

In response to diabetes mellitus, skeletal muscle is negatively affected, as is evident by reduced contractile force production, increased muscle fatigability, and increased levels of oxidative stress biomarkers. Apocynin is a widely used NADPH oxidase inhibitor, with antioxidant and anti-inflammatory potential. It has been effective for amelioration of a variety of disorders, including diabetic complications. Therefore, the present study was conducted to evaluate the effects and action mechanisms of apocynin in slow- and fast-twitch diabetic rat muscles. Male Wistar rats were rendered diabetic by applying intraperitoneally a single dose of streptozotocin (45 mg/kg). Apocynin treatment (3 mg/kg/day) was administered over 8 weeks. Fasting blood glucose (FBG), insulin tolerance and body weight gain were measured. Both slow (soleus) and fast (extensor digitorum longus, EDL) skeletal muscles were used for muscle function evaluation, oxidative stress markers, and evaluating gene expression using qRT-PCR. Treatment with apocynin significantly reduced FBG levels and enhanced insulin tolerance. Apocynin also prevented muscle contractile dysfunction in EDL muscle but had no significant effect on this parameter in soleus muscles. However, in both types of muscles, apocynin mitigated the oxidative stress by decreasing ROS levels and increasing total glutathione levels and redox state. Concomitantly, apocynin also statistically enhanced Nrf-2 and GLU4 mRNA expression and downregulated NOX2, NOX4, and NF-κB mRNA. Collectively, apocynin exhibits properties myoprotective in diabetic animals. These findings indicate that apocynin predominantly acts as an antioxidant in fast-twitch and slow-twitch muscles but has differential impact on contractile function.

## 1. Introduction

Diabetes mellitus (DM) is a group of metabolic disorders that results from insufficient insulin secretion or inappropriate insulin signal transduction, which establishes a state of hyperglycemia [1]. This disease has multisystemic manifestations. A common complication of any diabetes form (Type 1 or Type 2) is a failure to preserve muscle mass and function, a condition referred as diabetic myopathy [2,3]. Consequently, reduction in muscle mass is usually associated with decrease in muscle strength and quality, lower endurance, and contractile abnormalities resulting in muscle weakness, fatigue, and exercise intolerance [4,5]. Moreover, because skeletal muscle is the most important tissue for insulin-stimulated glucose disposal, an impaired muscle metabolism has been linked to mitochondrial dysfunction, inflammation, lipotoxicity, and insulin resistance [6].

Skeletal muscle is a heterogeneous tissue, due to the presence of two distinct populations of muscle fibers called “slow-twitch” (type I) and “fast-twitch” (type II), which display marked differences in contraction physiology, metabolic activity, and genetics. The muscle fiber-type composition can provide differential susceptibility to certain muscle diseases. In this sense, the response to hyperglycemia stimuli and diabetes may differ considerably from muscle to muscle with distinct fiber-type distribution [7]. Thus, illuminating the fiber-type-specific effects may provide important insights for discovering novel therapeutic strategies and delay complications from diabetes.

Oxidative stress represents a central factor linked to the pathogenesis of diabetic complications. It is caused by an excessive production of reactive oxygen species (ROS) that the antioxidative systems of cells cannot effectively counteract, which triggers a redox imbalance [3,8]. Oxidative stress is a major upstream event for diabetes complications as well as insulin resistance development due to inducing multiple pathophysiologic pathways [9]. Specifically in skeletal muscle, high levels of ROS wreak havoc within the tissue, leading to alterations in insulin signaling, lipotoxicity, mitochondrial dysfunction, and activation of inflammatory roads, promoting muscle dysfunction [10,11], as well as a modification of expression of genes that participate in antioxidant and metabolic defense [8]. In skeletal muscle, nicotinamide adenine dinucleotide phosphate (NADPH) oxidases (NOXs) enzymes are key producers of ROS and protagonists of redox homeostasis [12,13]. However, strong evidence suggests that NOX-generated ROS are a major contributor to oxidative damage in pathologic conditions, such as diabetes [14,15,16], and in muscle abnormalities in other settings [11,17]. The family of NOX enzymes consists of 7 members, NOX1–NOX5 and Duox1–2. Of those members, there are two isoforms of NOX present in skeletal muscle (NOX2 and NOX4), associated with sarcoplasmic reticulum and sarcolemma [12,13]. These NOX are specialized to produce certain kinds of ROS. While NOX2 produces •O2^−^, NOX4 directly forms hydrogen peroxide [13]. Studies of NOX enzymes have suggested connections between the increased NOX activity and expression of specific NOX members in diabetes. For example, an upregulated expression and activity of NOX2 has been found in diabetic hearts and is associated with several detrimental processes, including contractile dysfunction and cell death [16]. On the other hand, there is also evidence that increased NOX2 expression concomitant with ROS production contributes to skeletal muscle insulin resistance induced by a high fat diet [11]. However, the exact role of NOX-derived ROS in diabetes-induced muscle dysfunction remains unclear.

Apocynin is a natural organic compound structurally related to vanillin found in plant sources [18]. It has been proven to be an efficient NOX inhibitor in many cell and animal models [19,20,21]. It has been widely used for research purposes and for its therapeutic potential in a variety of disorders, such as diabetic complications [18]. It was found that apocynin restores serum antioxidant enzyme activities catalase and SOD in diabetic rats [22]. We recently reported that apocynin improved insulin sensitivity, attenuating oxidative stress and preserving mitochondrial function in the heart muscle of streptozotocin-induced diabetic rats (STZ) [21]. Regarding skeletal muscle, it was shown that the chronic administration of apocynin into HFD-fed mice significantly ameliorated the limited exercise capacity as well as mitochondrial dysfunction in skeletal muscle [10]. Additionally, the protective effect of apocynin was shown in diabetic nephropathy, diabetic endothelial dysfunction, and diabetic cardiomyopathy [18]. Nevertheless, its utility in myopathy diabetic remains to be determined. Thus, we hypothesize that apocynin could mediate an attenuation of the muscle metabolic and functional defects found in diabetes. The present work aimed to evaluate the effect of apocynin on muscle function, oxidative stress markers, and the expression of genes that participate in the antioxidant and metabolic response in the fast and slow muscles of STZ-induced diabetic rats.

## 2. Materials and Methods

### 2.1. Animals

The experiments were carried out with male Wistar albino rats weighing 230–250 g at the beginning of the experiments. The rats were kept in a specific pathogen-free environment on a 12-h light–dark cycle and maintained in a temperature range (22 ± 2 °C). Animals were provided a standard rat chow and water ad libitum. All experimental protocols and use of animals were performed in accordance with the Mexican regulations for use and care of laboratory animals (NOM-062-ZOO-1999) and with prior approval from the Institutional Committee for Use of Animals of the Universidad de Guanajuato (Code-CIBIUG-P06-2021).

### 2.2. Experimental Design

The animals were randomly divided into the following groups: normoglycemic rats (control), non-treated diabetic rats (diabetes), and diabetic rats treated with apocynin (diabetes + apocynin); *n* = 6 in each group. Diabetes was induced by a single streptozotocin (STZ) injection (45 mg/kg body weight) (Sigma-Aldrich, St Louis, MO, USA) that was freshly dissolved in a citrate buffer (0.5 M, pH 4.5), and control rats (normoglycemic) received a citrate buffer injection, intraperitoneally, instead of STZ. Treatment for eight weeks with apocynin (Tocris Bioscience) started 1 week after STZ, by intraperitoneal injection of 3 mg/kg/day. Animals in the control groups were managed similarly to those in the apocynin-treated group. Variations in body weight and FBG were evaluated every week. All groups were evaluated during the same period. Once treatment was complete, the animals of all groups were fasted for 8 h and euthanized using cervical dislocation. Skeletal muscle samples from EDL and soleus were immediately dissected from both the left and right hind limbs in all animals. Isometric tension measurements were performed using fresh muscles, and the remaining muscles were stored at −80 °C until processed by the glutathione assay, to measure ROS levels and to evaluate mRNA expression levels by real-time RT-qPCR.

### 2.3. Insulin Tolerance Test

At the end of experimental protocol, insulin tolerance test (ITT) was evaluated in all groups. Rats were fasted for 10 h, and basal blood glucose concentration was determined with blood from the tail tip using a glucometer (Accu-Chek Performa, Roche, Indianapolis, IN, USA). Blood glucose levels were determined at 0, 30, 60, 90, and 120 min after an intraperitoneal insulin injection (0.75U insulin/kg body weight). Blood glucose response to ITT was calculated as area under the curve (AUC-ITT) of the blood glucose excursion for 120 min after insulin injection and correlated with the corresponding fasting values according to the mathematical TAI model [23].

### 2.4. Isometric Tension Measurements

EDL and soleus muscles isolated from a left hindlimb (*n* = 6 in each group) were placed in a recording chamber for isometric tension measurements, with the proximal end attached to the bottom of the chamber and the distal end to the hook of an optical transducer, immersed in a physiological (Krebs-Ringer) solution (118 mM NaCl, 4.75 mM KCl, 1.18 mM MgSO_4_, 24.8 mM NaHCO_3_, 1.18 mM KH_2_PO_4_, 10 mM glucose, and 2.54 mM CaCl_2_) and carbogen gas (95% O_2_ and 5% CO_2_). The muscle was stretched to 1.3 times its resting length and left to perfuse in the physiological solution for 10 min before recording the isometric tension; the experiment was performed at a temperature of 25 ± 1 °C. For maximum and total isometric twitch tension measurements, the recording chamber was connected to an optical force transducer, which through an amplifier and an analog–digital interface (World Precision Instruments, Sarasota, FL, USA) allowed acquiring the tension generated by the muscle in a computer, using MDAC software (World precision instruments, Sarasota, FL, USA). Muscles were activated by supramaximal stimulation via platinum electrodes placed in a parallel direction to the muscle’s longitudinal axis. Two platinum electrodes were placed inside the recording chamber, which was connected to a stimulus isolation unit and an electric stimulator (Grass). To apply the protocol to fatigue induction, the muscle was repeatedly stimulated using an electric current to produce multiple isometric contractions over a period of time by applying 100 V pulses, 300 ms in duration, at the frequency of 45 Hz for soleus muscle and 50 Hz for EDL muscle; the electrical stimulation was generated until the muscle was fatigued (~70% reduction in the initial strength). The analyzed parameters were: (1) fatigue resistance measurement (time); (2) maximal tension (from basal line to the amplitude peak); and (3) the total tension measured by obtaining the area under the tension–time curve.

### 2.5. Measurement of Reactive Oxygen Species Levels

Levels of ROS were determined in muscle tissue by using the cell permeable fluorescent probe 2′,7′-dichlorodihydrofluorescein diacetate (H_2_DCFDA) according to Bravo-Sánchez et al. [21]. A total of 0.5 mg of protein was placed in 2 mL of buffer containing 100 mM KCl, 10 mM HEPES, 3 mM KH_2_PO_4_, and 3 mM MgCl_2_ (pH 7.4) and incubated with 12.5 µM of H_2_DCFDA for 15 min in an ice bath under constant shaking. Changes in fluorescence were recorded at 0 and 60 min at extinction 485 nm and emission 520 nm wavelengths in a spectrofluorophotometer (Shimadzu RF-5301PC, Kyoto, Japan).

### 2.6. Determination of Glutathione Status 

To determine the amount of glutathione present in EDL and soleus muscles, reduced glutathione (GSH) and oxidized glutathione (GSSG) were measured by the method of Rahman et al. [24].

### 2.7. Total RNA Extraction and Real-Time RT-qPCR for mRNA Expression Analyzes

Total RNA was isolated from muscle samples (EDL and soleus), which were immersed and homogenized in TRIzol (TRI Reagent, Sigma Aldrich, Saint Louis, MO, USA), using the method described by Chomczynski and Sacchi [25], with minor modifications. RNA quality and quantity were analyzed spectrophotometrically at optical densities at 260/280 ratio using the BioPhotometer (Eppendorf, Hamburg, Germany). Complementary DNA (cDNA) was synthesized from 2 μg of RNA using a cDNA synthesis kit (QIAGEN, Hilden, Germany), according to the manufacturer’s instructions. Quantitative reverse transcription polymerase chain reactions (qRT-PCR) were performed on a QuantStudio 3 Real-Time PCR System (Applied Biosystems, ThermoFischer, CA, USA), using the QuantiFast SYBR Green PCR Kit (QIAGEN, Hilden, Germany). Primer sequences were designed using information contained in the public data base in the Gene Bank of the National Center for Biotechnology Information. The sequences of the PCR primers used are shown in Table 1. The target genes expression was evaluated by the relative quantification method using the comparative delta–delta cycle threshold (ΔΔCT) method [26], with the endogenous housekeeping gene 18s as an internal control. 

### 2.8. Statistical Analysis

All statistical tests were carried out by using GraphPad Prism™ software version 6 (GraphPad Software Inc., San Diego, CA, USA). One-way analysis of variance (ANOVA) followed by Tukey’s multiple comparison test were used to analyze the data. The results were expressed as mean ± standard error of the mean (SEM), and the level of statistical significance was set at * *P* < 0.05. 

## 3. Results

### 3.1. Effect of Apocynin Treatment on Fasting Blood Glucose, Body Weight, and Insulin Tolerance in Diabetes

**Figure 1:**
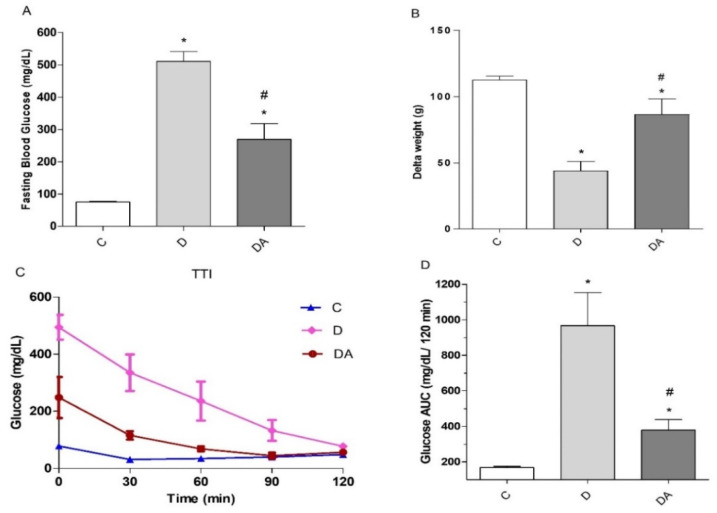
Effect of apocynin on (**A**) fasting blood glucose (FBG), (**B**) body weight changes (delta), and insulin tolerance test illustrated as (**C**) glucose levels pre- and post-insulin injection and (**D**) blood glucose expressed as total area under the curve (AUC). C = control group; D = diabetic group; DA = diabetes + apocynin group. Data are presented as the mean ± SEM (*n* = 6 per group). * *P* < 0.05 vs. C group. # *P* < 0.05 vs. D group.

First, we evaluated the potential effects of the NOX inhibitor apocynin on fasting blood glucose levels (FBG) (Figure 1A). FBG levels were remarkably higher in the diabetes group (515.65 ± 20.14 mg/dL) compared to the control group (75.30 ± 2.07 mg/dL; *P* < 0.001) (Figure 1A), thereby confirming the diabetic condition. After eight weeks of apocynin treatment, FBG levels in the diabetes + apocynin group were significantly lower than in the diabetes group, dropping by as much as 48% (*P* < 0.01), but they did not reach normal values (*P* < 0.01). Regarding body weight (Figure 1B), a notable decrease in weight gain (delta) was observed at the end of the experimental protocol in the diabetes group (42.50 ± 7.07 g; *P* < 0.05) compared to the control group (112.40 ± 2.92 g; *P* < 0.05), which is a hallmark feature of the pathology. However, intervention with apocynin prevented this effect, based on an observed significant improvement in weight gain in the diabetes + apocynin group (91.00 ± 11.59 g; *P* < 0.01) compared to the diabetes group. Meanwhile, insulin sensibility was assessed using the insulin tolerance test (ITT) performed at the end of the 8 weeks of apocynin administration. For the test, insulin was injected i.p., immediately after measuring basal fasting blood glucose levels (t = 0), and we measured blood glucose levels after 30, 60, and 120 min. Compared with the control group, the diabetes group showed a significant decrease in insulin sensitivity (*P* < 0.05). Changes in glucose induced by insulin injection were more evident and lasting in rats treated with apocynin (diabetes + apocynin group) (*P* < 0.01; Figure 1C). In Figure 1D, the ITT-AUC (Area Under the Curve for the ITT responses) was plotted for the three groups. Thus, apocynin also improved insulin tolerance in diabetic rats, as shown by significant decreases in glucose levels and the area under the ITT curve in the diabetes + apocynin group compared with the diabetes group (*P* < 0.01) (Figure 1D).

### 3.2. Effects of Apocynin on Contractile Properties and Fatigue of Slow-Twitch and Fast-Twitch Muscles

**Figure 2:**
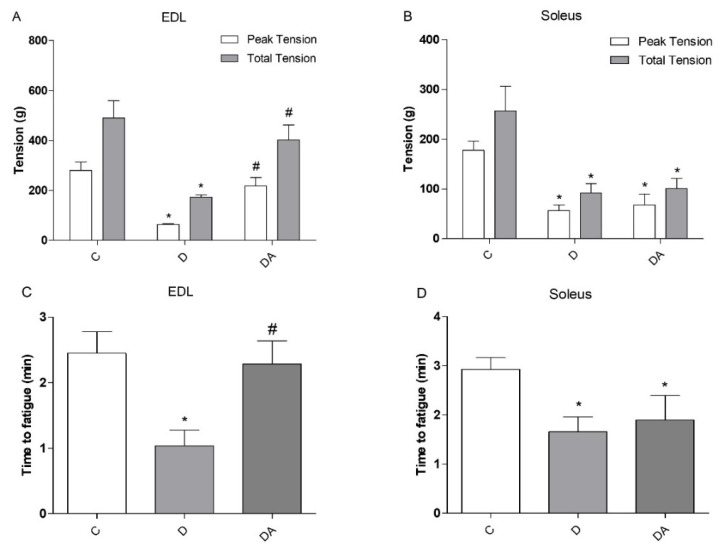
Effect of apocynin on maximum tension, total tension, and time of resistance to fatigue of slow and fast skeletal muscle of diabetic rats. (**A**) Maximum and total tension in EDL muscle, (**B**) maximum and total tension of the soleus muscle, (**C**) fatigue resistance time for EDL muscle, and (**D**) fatigue resistance time for soleus muscle. C = control group; D = diabetic group; DA = diabetes + apocynin group. Data are presented as the mean ± SEM (*n* = 6 per group). * *P* < 0.05 vs. C group. # *P* < 0.05 vs. D group.

To analyze the effect of apocynin on muscle function, isolated whole fast-twitch EDL and slow-twitch soleus muscles were fatigued by repeated tetanic stimulation while measuring contractile function. The time of resistance to fatigue was measured, as well as the maximum and total tension generated by the two types of muscles of the different experimental groups (Figure 2). Data indicated that muscle function from diabetic rats was compromised, which was evidenced by the decreased total tension and peak tension in EDL (Figure 2A) and soleus (Figure 2B) muscles and was accompanied by a markedly faster decline in force during fatiguing stimulation compared to the control group (Figure 2C,D). However, the treatment with apocynin in diabetic rats contributed to a significant increase in the time of resistance to fatigue (54.48%; *P* < 0.05) and improved maximum (70.64%; *P* < 0.05) and total (56.93%; *P* < 0.05) muscle tension compared to the diabetes group, but no differences were observed in soleus muscle. These results show that apocynin improves muscle function; however, the magnitude of effects was greater in EDL muscle than soleus muscle.

### 3.3. Apocynin Reduces the Levels of Reactive Oxygen Species in Fast and Slow Diabetic Skeletal Muscles

**Figure 3:**
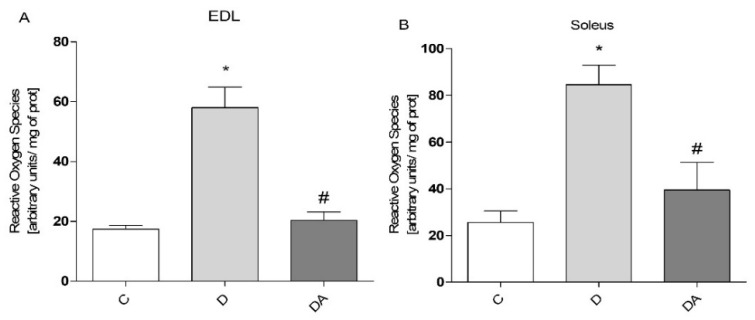
Effect of apocynin on levels of reactive oxygen species (ROS) in both EDL (**A**) and soleus (**B**) muscles. Apocynin significantly reduced ROS levels in both muscles of diabetic rats. C = control group; D = diabetic group; DA = diabetes + apocynin group. Data are presented as the mean ± standard error from 6 rats per group. * *P* < 0.05 vs. C group. # *P* < 0.05 vs. D group.

To explore whether apocynin has influence on ROS production, ROS levels in EDL muscle (Figure 3A) and soleus muscle (Figure 3B) of the different experimental groups were measured. As shown in Figure 3, we observed ROS accumulation in both muscles in response to diabetes compared to the control group, observing a higher level of ROS in soleus muscle than in EDL muscle (74.86% and 65.75%, respectively) under this condition (Figure 3A, B). In line with its NOX inhibitor role, the effect of apocynin was a significantly lower ROS generation (diabetes + apocynin group) in both EDL and soleus muscles (65.02% and 53.01%, respectively) compared to the diabetes group. Together, these results suggest that apocynin modulates diabetes-induced ROS production in both types of skeletal muscle.

### 3.4. Apocynin Improved Glutathione Redox Status in Fast and Slow Skeletal Muscle in Diabetic Rats

**Figure 4:**
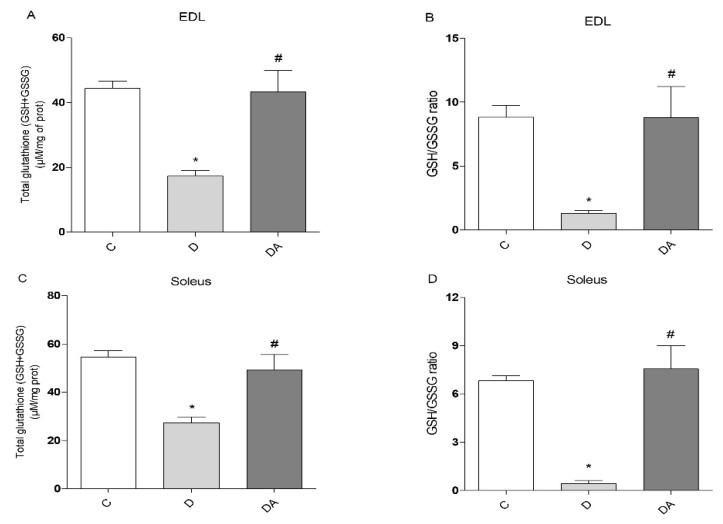
Effects of apocynin on glutathione redox status in skeletal muscle of diabetic rats. Total glutathione (GSH + GSSG) (**A**) and the redox ratio (GSH/GSSG) (**B**) in EDL muscles. (**C**) Total glutathione (GSH + GSSG) levels and (**D**) the redox ratio (GSH/GSSG) in soleus muscles. C = control group; D = diabetic group; DA = diabetes + apocynin group. Data are presented as the mean ± SEM (*n* = 6 per group). * *P* < 0.05 vs. C group. **#** *P* < 0.05 vs. D group.

To determine whether lower ROS levels caused by apocynin in diabetic rat muscles were parallel to oxidative status changes, the glutathione defense system was evaluated in the EDL (Figure 4A,B) and soleus muscles (Figure 4C,D). Reduced concentrations of total glutathione (TGSH) were observed in EDL (Figure 4A) and soleus muscles (Figure 4C) from the diabetes group (60.83% and 49.94%, respectively), compared to control rats (*P* < 0.05). The redox status of glutathione (GSH/GSSG ratio) also changed considerably in diabetic rats when compared to the control group, and the GSH/GSSG ratio in EDL (Figure 4B) and soleus muscles (Figure 4D) of the diabetes group was significantly lower, at 85.15% and 93.70%, respectively. However, the total GSH levels in EDL (Figure 4A) and soleus muscles (Figure 4C) were significantly increased (59.87% and 44.55%, respectively) (*P* < 0.05) in the diabetes + apocynin group when compared to the diabetes group. Consistently, apocynin also improved the redox status, with a significantly higher GSH/GSSG ratio in EDL (78.38%; *P* < 0.05) and soleus muscle (96.73%) in the diabetes + apocynin group compared to the diabetes group (Figure 4B,D); these parameters were similar to control levels in both muscles.

### 3.5. Effects of Apocynin on NOX2 and NOX4 Expression in Fast- and Slow-Twitch Skeletal Muscle in Diabetic Rats

**Figure 5:**
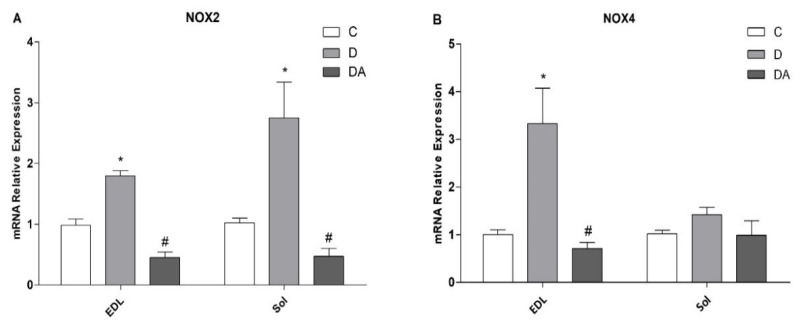
Effect of apocynin on mRNA expression levels of NOX2 (**A**) and NOX4 (**B**) in both EDL and soleus muscle. C = control group; D = diabetic group; DA = diabetes + apocynin group. Data are presented as the mean ± SEM (*n* = 6 per group). * *P* < 0.05 vs. C group. # *P* < 0.05 vs. D group. *P* < 0.05 in EDL vs. soleus muscle.

The expression of mRNA of NOX2 and NOX4 enzymes in fast- (EDL) and slow (soleus)-twitch skeletal muscle was quantified by RT-qPCR (Figure 5). As shown in Figure 5A, NOX2 mRNA levels were upregulated in both muscles from the diabetes group, whereas NOX4 only was higher in EDL muscle (Figure 5B) compared to control rats, while in soleus muscle remained unchanged compared to all groups (Figure 5B). As expected, treatment with apocynin reduced the level of expression of NOX2 in both muscles (Figure 5A), and a lower-level expression of NOX4 was detected in EDL muscle compared to the diabetes group (Figure 5B).

### 3.6. Effect of Apocynin on Gene Expression of Nrf2, NF-ҡβ, and GLUT4 in the Fast and Slow Muscles of Rats with Diabetes

**Figure 6:**
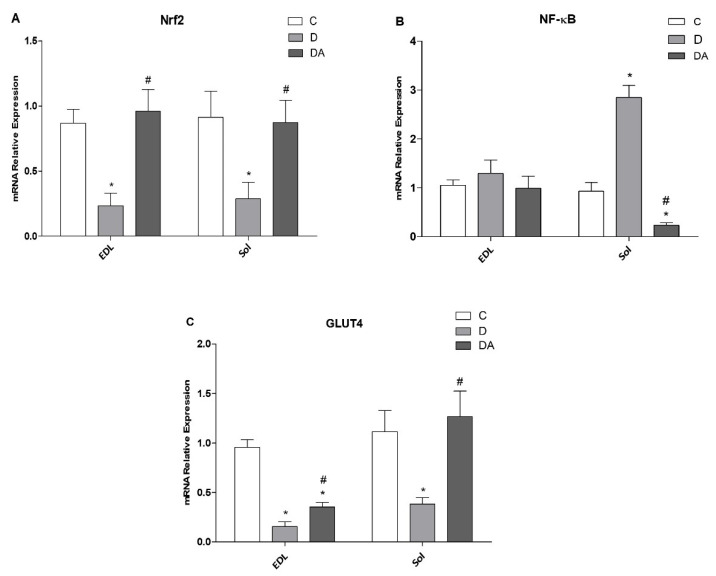
Effects of apocynin on expression of Nrf2 (**A**), NF-kb (**B**), and GLUT4 (**C**) mRNA in EDL (fast-twitch) and soleus (slow-twitch) muscles. C = control group; D = diabetic group; DA = diabetes + apocynin group. Data are presented as the mean ± SEM (*n* = 6 per group). * *P* < 0.05 vs. C group. # *P* < 0.05 vs. D group. Nuclear factor erythroid 2-related factor 2 (Nrf2), Nuclear factor-kappa B (NF-κB), glucose transporter 4 (GLUT4). *P* < 0.05 in EDL vs. soleus muscle.

As shown in Figure 6, in diabetic rats, the mRNA expression levels of Nrf-2 (Figure 6A) and GLUT4 (Figure 6C) were statistically downregulated in EDL (73.09% and 83.39%, respectively; *P* < 0.01) and soleus muscle (57.26% and 70.51%, respectively; *P* < 0.05) compared to the control group, while treatment with apocynin significantly enhanced Nrf2 and GLUT4 expressions in both EDL (75.66%) and soleus muscles (69.54%) compared to the diabetes group. Interestingly, GLUT4 mRNA levels were higher in soleus muscle than in EDL in the diabetes + apocynin group. However, marked differences between EDL and soleus were found in mRNA levels of NF-ҡβ (Figure 6B); it was upregulated only in soleus muscle from the diabetes group, exhibiting 67.37% (*P* < 0.01) compared to those from the control group. Meanwhile, in apocynin-treated diabetic rats, NF-ҡβ was prominently downregulated in soleus muscles. Oppositely, there was no difference in mRNA expression of NF-ҡβ in EDL muscle compared to all groups (Figure 6B).

## 4. Discussion

Diabetic myopathy is a complication of diabetes characterized by impairments of structural, functional, and metabolic capacities in skeletal muscle, and ROS overproduction and oxidative stress have an essential role in this condition [2,27]. Advances in the comprehension of diabetes have led to the search for new tools that are aimed at precisely deciphering and targeting ROS-triggered pathways to prevent oxidative damage and its impact to skeletal muscle tissues; accordingly, in this study we investigated the effect of apocynin, an inhibitor of NADPH oxidase [28]. The results of the current study demonstrate that treatment with apocynin alleviates negative diabetic effects in fast and slow skeletal muscles. Additionally, our data suggest a key role of apocynin treatment of Nrf2 and NF-κβ expression in the regulation of diabetes-induced oxidative stress in skeletal muscle.

STZ can mimic the metabolic features of DM, with symptoms that resemble the natural disease process [29]. The present study shows that levels of fasting blood glucose and insulin resistance were significantly increased in rats with STZ-induced diabetes. The diabetogenic properties of STZ are due to the selective destruction of β-cells and insulin deficiency, which lead to hyperglycemia. In this condition, the insulin deficiency leads to proteins being degraded to provide amino acids for gluconeogenesis, resulting in the loss of muscle mass and weight loss [29], which can explain the decline and low body weight gain in diabetic animals compared to healthy rats. Interestingly, these critical biomarkers were attenuated in animals treated with apocynin in comparison to untreated diabetic animals (Figure 1). Results of the present study reinforce the beneficial effects of apocynin in improving glucose metabolism [21,30]. It has been demonstrated that inhibition of renal gluconeogenesis is involved in apocynin hypoglycemic action in diabetic rabbits [30]. Similar findings have shown that apocynin significantly reduced hyperglycemia, hyperinsulinemia, and dyslipidemia by improving insulin sensitivity in high-fat-diet (HFD)-induced obese mice as well [31]. Additionally, apocynin treatment for 8 weeks prevented β-cell apoptosis and ameliorated insulin deficiency in rats with accumulation of plasma advanced oxidation protein products, controlling the advance of diabetes [32]. Thus, these findings suggest that apocynin has antidiabetic activity.

Given that insulin exerts anabolic effects for muscle cells, insufficiency of insulin action and prolonged hyperglycemia result in muscle wasting, altered metabolic capacity, and reductions in muscle function [3]. In this study, muscle dysfunction was evidenced by reduced contractile force and increased fatigability in EDL and soleus muscles, which represent two muscle types that are different in their metabolic and contractile properties (i.e., fast/glycolytic and slow/oxidative, respectively) [33]. These alterations often are accompanied by mitochondrial dysfunction and ultimately lead to exercise intolerance in diabetes [10]. Moreover, there is evidence linking a disrupted muscle insulin signaling to excess ROS production and elevated markers of oxidative stress, which are significant metabolic abnormalities implicated in muscle fatigue and reduced contractile force [3], as observed in EDL and soleus muscles in our study. In parallel, GSSG levels markedly increased, with a significant decline in the GSH/GSSG ratio, and a concomitant decrease in levels of total GSH both in EDL and soleus muscles, which is indicative of an oxidative state [34] and believed to account for proteins dysfunction required for proper muscle contraction, by altering the redox status in muscle cells [35]. However, this study has shown that treatment with apocynin can prevent consequences of diabetic myopathy. In our results, apocynin improved the muscle function in EDL muscle by increasing muscle tension and promoting resistance to fatigue in the diabetic rats treated with this agent; however, these parameters remained unchanged in the soleus muscle. Consistent with previous reports, chronic administration of apocynin into HFD-fed mice improved exercise intolerance and ameliorated mitochondrial dysfunction in skeletal muscle, which are significant metabolic alterations implicated in diabetic muscle as well [36]. Moreover, research in vitro and in vivo studies have reported significant effects of apocynin against diabetic complications through its ability to limit ROS production and due to its antioxidative effects [18,28]. In this work, despite the differential effects of apocynin on contractile function between EDL and soleus muscles, apocynin significantly reduced ROS levels and promoted both enhancement of GSH levels and consequently the elevation of GSH/GSSG ratio in both types of muscles compared to the diabetic group, indicating that apocynin effectively boosts antioxidant capacity and ROS detoxification in muscle cells, as it has been confirmed in other tissues under this condition [18,21,22].

Expression and activation of NOX proteins particularly increases under conditions of acute and chronic stress, such as hyperglycemia, leading to a critical increase in NOX-derived ROS, causing oxidative stress and cellular damage [14]. Skeletal muscle is known to express two of the NOX isoforms, NOX2 and NOX4. NOX2 and its regulatory subunits and NOX4 are present in the sarcolemma, sarcoplasmic reticulum, and T tubules of muscle fibers. Furthermore, NOX activity and expression differ according to the skeletal muscle fiber type, as well as antioxidant defense [37]. In the present report, we have shown that diabetes was able to increase the mRNA expression levels of NOX2 in both types of muscles and NOX4 only in the EDL fibers. In addition, NOX2 expression was higher in soleus when compared to EDL. Consistent with the involvement of NOX2, an upregulated expression and activity of NOX2 has been found in diabetic hearts and is associated with several detrimental processes, including contractile dysfunction and cell death [16]. Likewise, reports suggest that NOX2 mediates skeletal muscle insulin resistance induced by a high fat diet [11]. In contrast, downregulation of NOX2 in C2C12 cells prevented insulin resistance induced by high glucose or palmitate [11]. Similarly, studies by Bechara et al. [17] showed that muscle atrophy in rats with heart failure is associated with increased NOX2 mRNA, suggesting that this is the main isoform responsible for increased NOX activity under different conditions in skeletal muscle. Nevertheless, our results suggest that both NOX2 and NOX4 appear to be involved in diabetic muscles. Interestingly, all these alterations have been mitigated by apocynin. Apocynin’s action for NOX has been supported by studies reporting that it prevented translocation of the p47phox subunit to the plasma membrane, thus causing inhibition of NOX enzyme. Therefore, apocynin is extensively used to reveal the role of this enzyme in cell and experimental models [18,19,20]. In this work, markedly, apocynin modulated the mRNA expression of these NOXs as well. We showed that mRNA levels of NOX2 and NOX4 in the apocynin-treated diabetic group were downregulated and recover to control levels in both muscle types. Based on these facts, our findings point out the potential role of NOX enzymes in skeletal muscle impairment in diabetes, as is seen with other diabetes complications [14,16,18]. Moreover, these findings suggest that fast-twitch muscle is more sensitive to the unstable redox environment linked with diabetes at the transcriptional level of NOX enzymes compared to slow-twitch muscle.

On the other hand, both Nrf2 and NF-κB are key pathways regulating the fine balance of cellular redox status and responses to stress and inflammation in this disease [38,39]. Our study showed that Nrf2 mRNA expression levels were prominently downregulated in both types of muscles from diabetic animals compared to healthy animals. Concomitantly, we found that the expression of NF-κB in response to enhanced ROS production resulted in differential alteration in a muscle-specific manner; NF-κB was upregulated in soleus muscles of diabetic rats, remaining unchanged in the diabetic EDL muscle when compared to the control group. Despite these differences, the expression of these genes in response to increased ROS levels may have physiological and molecular implications for controlling redox homeostasis [38]. The negative effect of Nrf2 downregulation may amplify inflammatory responses by inducing the expression of NF-κB, IL-1ß, and TNF-α [40] along with an increased oxidative and nitrosative stress [38,39]. In this context, NF-κB is a transcription factor that regulates expression of many kinds of cytokines and inflammatory proteins in oxidative environments, and the increased NF-κB signaling decreases insulin action and promotes insulin resistance in the liver and whole body [41]. In line with this fact, Nrf2 deletion may lead to hepatic insulin resistance by activation of NF-κB pathway [42]. In skeletal muscle, activation of NF-κB transcriptional activity apparently serves a dual function by inducing both fast-twitch fiber atrophy and slow-twitch fiber degeneration [43]. Meanwhile, the ability to scavenge ROS and handle oxidative stress is dramatically reduced in the muscles of Nrf2 KO animals [44]. Nrf2, is a master transcriptional factor of antioxidative defense systems, and accumulated evidence has suggested that activation and upregulated expression of Nrf2 may have therapeutic potential in diabetes complications [39,45]. Interestingly, our study showed that apocynin can upregulate and restore the balance in expression levels of Nrf2 due the pathological changes in diabetic muscles. Likewise, protective effects of apocynin have been attributed to the activation of Nrf2 by reducing the level of proinflammatory cytokines in an experimental murine colitis model [46]. In addition, NF-κB inhibition seems to be responsible for protective activity of apocynin, as there was reduction in mRNA expression of NF-κB in soleus muscle after apocynin treatment, approaching the control group. These findings are in agreement with previous reports in which it has been already reported that apocynin may exert other effects beside its ability to inhibit NOX [41]. Furthermore, our result is in accord with a study conducted by Pan and Quian, who used apocynin in a rat model of cerebral infarction and found that apocynin treatment significantly decreased NF-κB mRNA expression [47]. Therefore, this finding may suggest that the suppression of Nrf2 expression and increased expression of NF-κB are involved in the pathogenesis of diabetic myopathy, and NOX could be an upstream mediator of these changes.

Additionally, studies in C2C12 skeletal muscle cells in examining NF-κB inhibition coincide with significantly elevated levels of GLUT4, resulting in increased glucose uptake [48]. Thus, the intent of the present study was to determine whether these metabolically induced alterations by diabetes in skeletal muscle may be regulated at least in part at the level of transcription of the GLUT4 gene. The possibility that muscles GLUT4 protein may be subject to regulation at the transcriptional level was suggested by Garvey et al. [49], who reported a significant decrease in GLUT4 mRNA in the quadriceps muscles of rats with STZ-induced diabetes. Similarly, the GLUT4 mRNA expression was significantly decreased in EDL as well as soleus muscles. Interestingly, these changes were prevented by apocynin as well; it increased GLUT4 mRNA expression in soleus muscle and, to a smaller extent, EDL muscle in diabetic rats treated with apocynin. In fact, we found that the mRNA levels of GLUT4 in soleus muscle recovered to control levels. Hence, these data provide evidence that GLUT4 expression in skeletal muscle is subject to regulation by apocynin in a muscle-type-specific manner.

Based on all the results presented in this study, our findings point out the potential role of NOX enzymes in diabetic myopathy. To our knowledge, this is the first study conducted to verify the effectiveness of apocynin in treating hyperglycemia-induced oxidative stress and skeletal muscle dysfunction in an animal model of diabetes. Apocynin’s protective effects are significant for glucose metabolism and skeletal muscle function through improving antioxidant status and reduction of oxidative stress metabolites. Moreover, these data suggest that apocynin prevents muscle damage by regulating NF-κB and Nrf2 pathways.

## 5. Conclusions

In summary, the present work exhibits the antidiabetic, antioxidant, and myoprotective benefits of apocynin in controlling diabetes in slow- and fast-twitch muscles. The results suggest that the protective effect of apocynin may be partly attributed to the inhibition of NOX-induced oxidative stress and improvement of homeostasis redox. Effects of apocynin may be closely related to attenuation of upregulation of NF-κB signaling and upregulation of Nrf2 and GLUT4 expression in skeletal muscle. This research reinforces the beneficial potential of antioxidants in preventing diabetic complications in skeletal muscle.

## Figures and Tables

**Table 1:** PCR primer sequences.

Gene	Forward	Reverse
NOX2	5′-CAATTCACACCATTGCACATC-3′	5′-CGAGTCACAGCCACATACAG-3′
NOX4	5′-TCCATCAAGCCAAGATTCTGAG-3′	5′-GGTTTCCAGTCATCCA-TAGAG-3′
Nrf2	5′-CACATCCAGACAGACACCAGT-3′	5′-CTACAAATG-GAATGTCTCTGC-3′
NF-κB	5′-ATGGCAGACGACGATCCTTTC-3′	5′-TGTTGACAGTG-TATATCTGTTG-3′
GLUT4	5′-TCCATCAAGCCAAGATTCTGAG-3′	5′-GGTTTCCAGTCATCCA-TAGAG-3′
18s	5′-GCAAATTACCCACTCCCGAC-3′	5′-CCGCTCCCAAGA TCCAACTA-3′

## Data Availability

The data used to support the findings of this study are included in the manuscript.
